# Farmers’ views and attitudes towards bovine tuberculosis and bluetongue in Northern Ireland using semi-structured interviews

**DOI:** 10.1186/s13620-026-00335-5

**Published:** 2026-02-19

**Authors:** H Bishop, MJH O’Hagan, J Gillespie, FD Menzies

**Affiliations:** Veterinary Epidemiology Unit, Department of Agriculture, Environment and Rural Affairs, 303 Airport Road West, Belfast, BT3 9ED Northern Ireland

**Keywords:** Bovine tuberculosis, Bluetongue, Attitudes, Behaviours, Disease control, Disease eradication, Policy

## Abstract

**Background:**

Understanding the attitude of farmers towards infectious diseases and government interventions is essential for understanding behavioural responses during outbreaks, compliance with control measures, and preventing and eradicating diseases.

**Objectives:**

This study aimed to improve the understanding of farmers’ attitudes towards bluetongue (BT) and bovine tuberculosis (bTB), which are key epizootic and endemic diseases in Northern Ireland, and provide insight into how this may affect disease control and eradication.

**Methodology:**

Short answer question slips, each containing a single question, relating to BT and bTB were presented on the Department of Agricultural, Environment and Rural Affairs stand at the Balmoral Show in May 2025. Visitors were encouraged to anonymously complete the questions. Simultaneously, visitors to the show were interviewed using semi-structured questionnaires on either BT or bTB. The conversations were recorded and transcribed. Grounded Theory was used to analyse transcripts.

**Results:**

A total of 141 question slips, each containing a single question, were completed. Of which, 99 questions related to bTB and 42 to BT. Interviews ranged between 1 and 3 minutes. There were 77 interviews: 46 for BT and 31 for bTB. Results show that stagnation in bTB eradication has led to frustration and disillusionment creating an environment fertile for blame, conspiracy theories and superstition. Respondents were aware of the limitations of bTB diagnostic tests. However, their confidence in a negative bTB skin test varied depending on the situation. Most farmers expressed concern about the risk of a BT incursion. However, the impact was perceived in terms of government-imposed control measures rather than the clinical impact of cases. Most farmers were supportive of movement restrictions. A small number of farmers expressed cynicism and highlighted the negative impact of movement restrictions.

**Conclusions:**

Information is available continuously through various platforms, but misinformation and disinformation is widespread. Combatting this, and disseminating accurate, timely information is increasingly challenging but necessary for eradication to be successful. Ascertaining the extent to which farmers’ confidence in the bTB skin test varies across different situations and how it impacts behaviour would be beneficial to disease control policy. Discussing BT usually elicited an acute, often abstract, fear from farmers compared to bTB where they focused on the financial burden and mental health impact of long-term control measures.

## Background

Understanding the attitude of farmers towards infectious diseases and government interventions is essential for understanding behavioural responses during outbreaks, compliance to policy control measures, and successfully preventing and eradicating diseases [3, 12]. However, attitudes towards infectious diseases are not uniform. They vary depending on the infectious disease in question, the epidemiological context of the disease, and the group surveyed. The current study was conducted to improve the understanding of farmers’ attitudes towards bluetongue (BT) and bovine tuberculosis (bTB). It also aimed to compare attitudes towards endemic versus epizootic diseases, providing insight into how this may affect disease control and eradication programmes. BT and bTB were selected as they represent key epizootic and endemic diseases in Northern Ireland (NI).

At the time of writing, NI has only had a single outbreak of BT, which concerned an imported heifer, in November 2018 [11, 15] but BT has never been considered to be established in the midge population of NI. However, the 2023 outbreak of BT serotype 3 (BTV-3) in the Netherlands with subsequent spread through mainland Europe and England has increased the risk of a BT incursion into NI. In the Netherlands, the impact of BTV-3 has been high with a significant increase in ruminant mortality [13, 20, 21] and decreased milk yield in dairy cows [20]. Due to the geographical proximity of infected countries and political ties between England and NI, the risk of a BT incursion into NI is moderate during the vector active season. Therefore, risk mitigation measures, such as a ban on importing animals from infected countries, is currently in place in NI. In light of this, BT was selected as an epizootic disease that is of current importance to both farmers and policy makers.

Unlike BT, bTB is endemic in NI. Every cattle herd in NI is tested annually using the comparative intradermal tuberculin test (CITT). In addition, there is active surveillance for bTB lesion at slaughter. When bTB is identified in a herd, often referred to as a breakdown, it is compulsory to slaughter all animals that have tested CITT positive. In addition, the whole herd is placed under mandatory animal movement restrictions. Breakdown herds are tested every 60 days until there are two consecutive whole herd tests that are negative. This can have a negative impact on animal welfare, business cash flow, profitability and farmer mental health. Despite decades of increasing bTB control measures, eradication remains elusive, which has had a detrimental impact on industry-government relationships [1, 17]. A Chief Veterinary Officer (CVO) review of bTB in 2024 aimed to cast a new light on bTB and design a new approach to its eradication in NI [7]. Therefore, bTB was selected as an endemic disease of particular interest to policy makers and industry.

## Methodology

The Department of Agriculture, Environment and Rural Affairs (DAERA) has a regular presence at the Balmoral Show, the largest annual agri-food event in NI. Farmers from across NI attend the Balmoral Show, thus providing an ideal opportunity for DAERA to explore their views on BTV and bTB. At the 2025 Balmoral Show, a series of printed, short answer question slips relating to BT and bTB, were made available at the DAERA veterinary stand. To encourage participation, each question slip contained a single question. There were 5 questions available on BT and 11 questions on bTB (see Appendix, Table 1). Visitors to the stand were encouraged by DAERA staff, who manned the stand continuously throughout the show, to select and complete one or more question slips and return it to a postbox. Those choosing to visit the DAERA stand usually do so because they have a query for DAERA staff. As such, they are often, but not exclusively, farmers or members of the agricultural community. The question slips were laid out on the stand in a manner that allowed visitors to peruse and select a question of interest to them. DAERA staff manning the stand could then use the selected question to facilitate conversations with visitors to the stand. Whilst not all DAERA staff on the stand are veterinary surgeons, all had received sufficient training to be able to engage visitors in conversations regarding bTB and BT. The responses to the question slips were analysed using counts and percentages. Confidence intervals were not calculated due to the low number of responses per question.

Simultaneously, over the first two days of the show, two DAERA staff explored the showground encouraging visitors to the show to participate in a short, semi-structured interview on either BT or bTB. Interviews were mostly conducted around the cattle and sheep sheds or the showing rings as these were good locations for identifying members of the farming community. The topic of the interview was determined by the interviewer; with interviewer 1 focusing on BT the first day and bTB the next day and vice versa for interviewer 2. Open questions were used to stimulate conversation. The conversations were recorded and transcribed. A Grounded Theory approach was used to analyse transcripts because this starts with the data and uses this to formulate a hypothesis rather than approaching the data with a predefined hypothesis [5, 22]. In line with this, coding was inductive with all codes derived from the data. Coding occurred in three stages; open, axial and selective coding. Open coding involved the transcripts being examined line-by-line and a code assigned. The codes and transcripts were reviewed every 3–4 manuscripts. During axial coding, the codes were mapped by considering cause-effect relationships and placed in hierarchies under defined categories. The final step, selective coding, considered the frequency and linking of categories and aimed to identify a core story. Interviewer 2 and a DAERA staff member, that was not involved in the interviews, were responsible for coding.

## Results

In total, there were 141 responses to the question slips, each containing a single question. Of these, 99 (70%) were on bTB and 42 (30%) on BT. The number of responses to each question varied from 0 to 30 (Appendix, Table 1). Due to the nature of the collection method, the number of individual respondents is unknown as visitors to the stand may have completed multiple question slips.

A total of 77 interviews were completed, 46 (60%) for BT and 31 (40%) for bTB (Appendix, Table 2). It was observed by both interviewers that people were more reluctant and more likely to decline to be interviewed about bTB compared to BT. Interestingly, this was often followed by superstitious responses such as “I don’t want to jinx myself”. However, on average, interviews for bTB were longer than BT suggesting that people have a greater knowledge and/or opinion on bTB (Appendix, Table 2). Most interviewees were male (91%, *n* = 70), over 50 years old (62%, *n* = 48) and had an education level up to or including A-Levels or their equivalent (71%, *n* = 53) (Appendix, Figs. 3A, 4A and 5A).

For the bTB interviews, most interviewees were beef farmers (*n* = 23, 74%), with a median herd size of 60 (range: 10–260), and a small number of participants were dairy farmers (*n* = 4, 13%), with a median herd size of 150 (range: 70–250). There was one interviewee with a mix of dairy and beef cattle, two interviewees working in other areas of the farming industry and one interviewee declined to answer this specific question.

For the BT interviews, there was an even split between sheep farmers (*n* = 13, 28%), with a median flock size of 135 (range: 20–200), beef farmers (*n* = 13, 28%), with a median herd size of 45 (range: 6-235), and mixed enterprises (*n* = 14, 30%), that had a median of 150 head of cattle and sheep (range: 30-1200). There was also a small number of interviewees that were dairy farmers (*n* = 6, 13%) with a median herd size of 190 (range: 70–420).

### Bovine tuberculosis

The number of responses to each question slip at the stand was very low (range: 0 to 30 per question). Therefore, care in interpretation is required. Firm conclusions cannot be reached based on the small sample sizes achieved for each question.

All respondents (*n* = 4 out of 4 responses) to the question slip “Do you feel able to make decision that will reduce the risk of a bTB breakdown in your herd?” said that they felt able to make these decisions. This is consistent with the findings of the interviews during which many farmers reported making positive changes to reduce their risk of a bTB herd breakdowns:

“I’ve become a closed herd, haven’t bought anything in the last three years.”

“I’ve hung the mineral blocks up on a rope. We thought they were licking them, the badgers.”

“Trying to keep round water troughs clean and cleared, trying to make sure that no other wildlife can enter the water trough to take drinks out of it or whatever.”

Most (75%, *n* = 6 out of 8 responses) respondents to the question slip stating, “If on-farm biosecurity advisory visits were available, would you be interested in participating?” said they would welcome such a visit. All respondents (*n* = 4 out of 4 responses) said that linking compensation with compliance to good biosecurity would encourage them to change their current biosecurity practices. The amount of additional compensation required per reactor varied depending on the complexity of the biosecurity measure and ranged from £0 additional compensation per reactor to isolate positive animals to £1000 additional compensation per reactor to only purchase from herds that had been free of bTB for 3 years. Similarly, most farmers (67%, *n* = 6 out of 9 responses) said that they would be willing to financially contribute to a wildlife intervention with culling (50%, *n* = 15 out of 30 responses) or Test and Vaccinate or Remove (TVR, 37%, *n* = 11 out of 30 responses) being more popular than vaccination alone (13%, *n* = 4 out of 30 responses).

However, it was clear from the interviews that the stagnation in bTB eradication over several decades despite ever increasing control measures has led to frustration:

“Well, we’re how many years with TB in the Northern Ireland now? I think twenty something years since it turned bad and we are worse off now than where we started.*”*

“They’re trying to eradicate TB, I don’t know how many years, 30, 40 years now. They’re worst off now than they were.”

and disillusionment.

“I don’t think it’s ever going to go away.”

“We’re still just kicking a can down the road. We’re not actually doing a lot about it.”

“I think most farmers, like myself, are just punched drunk with it all.”

This has created an environment fertile for blame,

“The last I heard was, we were at a meeting, and it was the Chief Veterinary Officer, blaming biosecurity on farms more than the wildlife side of things and that didn’t go down well. A lot of farmers would be trying their best to keep TB out.”

“Was it the veterinary practice? Or was it dormant then just came alive coz 6 months and its alive? Maybe it’s between the two injections? You performed them the wrong way around and you’re getting a different reading?”^1^

conspiracy theories and superstition.

“I’m just sceptical. I’ve never seen an animal sick with TB and I think it’s all rotten to the core. The department is a nice cushy job, so it’s very much in their interest to keep the thing going. I think TB is invented in places. There’s no TB at all, but it’s invented, to keep the workload up and keep somebody under pressure.”

“Well, I think at the minute their motivation is, it’s one way of culling numbers, culling herd numbers. You have to cull if they put you down with TB. They can cull you regardless, if you understand me.”

The sources of information reported on the question slips can be found in Appendix Fig. 6A. Against this backdrop, the “Review of Bovine TB” published by the CVO months earlier had little impact with all, bar one interviewee, being apparently unaware of its existence despite prompting from interviewer 2 [7].

Whilst the audience was split regarding their opinion of wildlife involvement in the transmission of bTB, there was consensus regarding the inadequacies of the current diagnostic tests available. The lack of progress in developing new, more accurate, less subjective tests added to the frustration of farmers.

“As well it’s like, the skin test is, what, 60 years old now and it’s still not fully 100% accurate.”

“Testing though would be the main thing I’m not too happy about, don’t think those skin tests are all that accurate so think there needs to be a change there.”

The challenges presented by imperfect and different test characteristics between the skin test, the blood test and postmortem examination, the subjectivity of ‘reading’ the skin test and fluctuations in an individual cow’s response to any given test over time has further fuelled their frustration and confusion

“But that’s why it’s a little bit, you can pass some tests and not pass all tests, you know, it’s strange.”

“You know what I mean, like, how all of a sudden, you just go down, loose a lot of cattle, and then all of a sudden you get through a clean test and you’re flying again.”

“I think maybe more investigations and improving the blood sampling for TB. The skin testing isn’t great. It can be inconclusive, up and down results.”

**Fig. 1:**
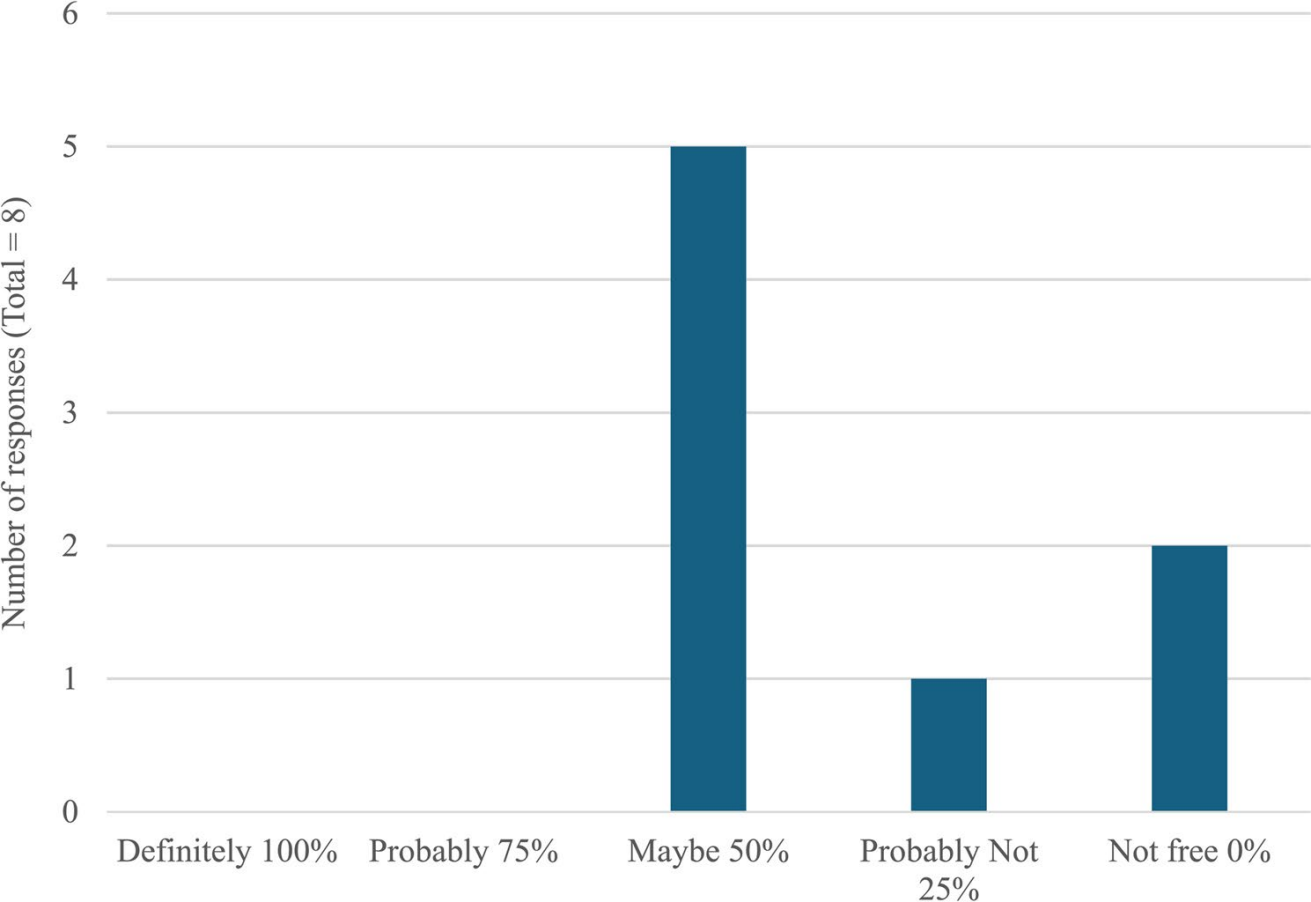
The probability assigned by respondents to the question; “If you are buying an animal from an officially bTB free herd that had a breakdown in the previous 12 months, what do you think the probability is that the herd is bTB free?” (*n* = 8 responses)

**Fig. 2:**
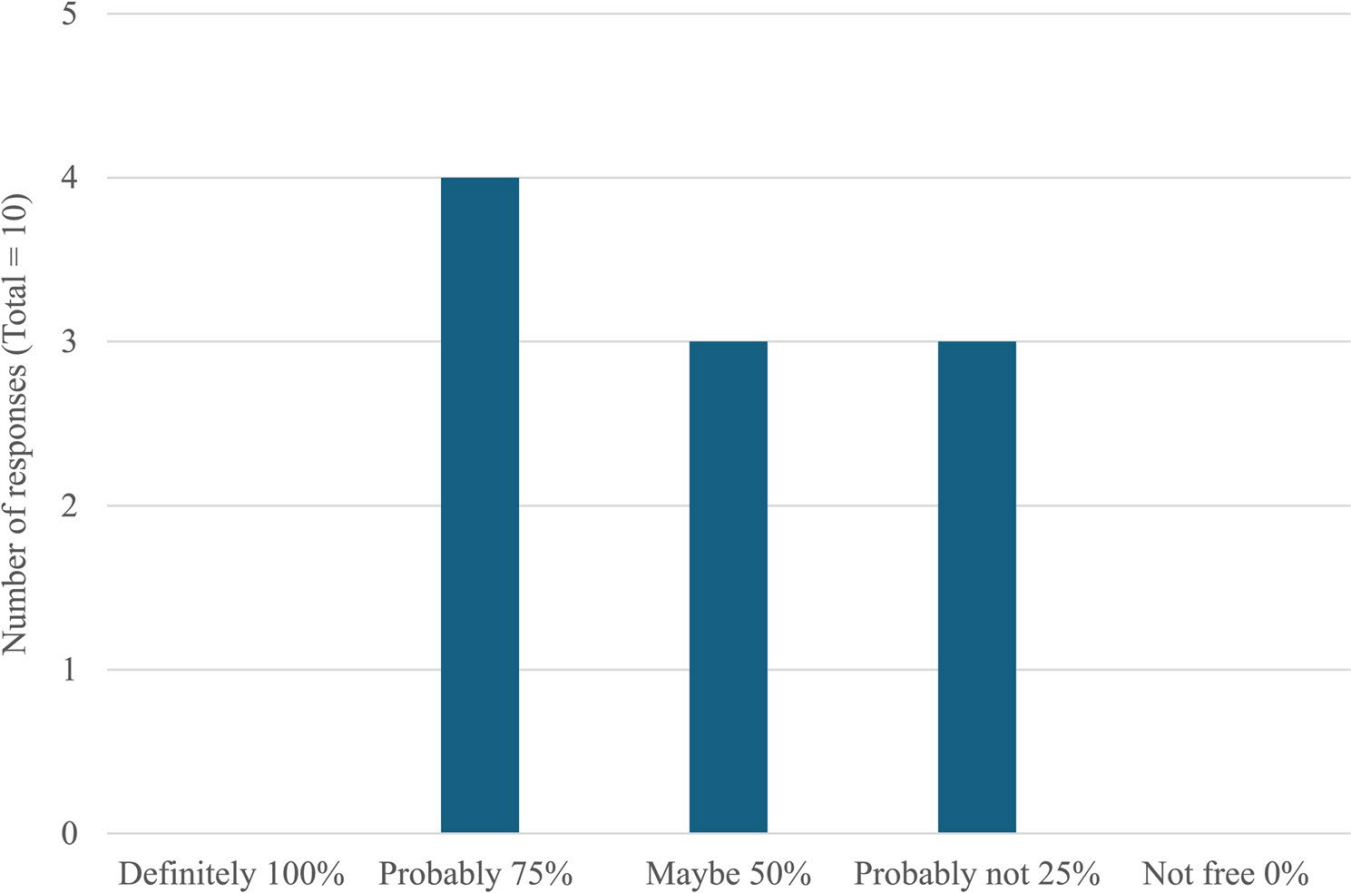
The probability assigned by respondents to the question; “If you have a bTB breakdown, then have 2 clear, whole herd tests and regain bTB freedom, what do you think the probability is that the herd is bTB free?” (*n* = 10 responses)

**Fig. 3:**
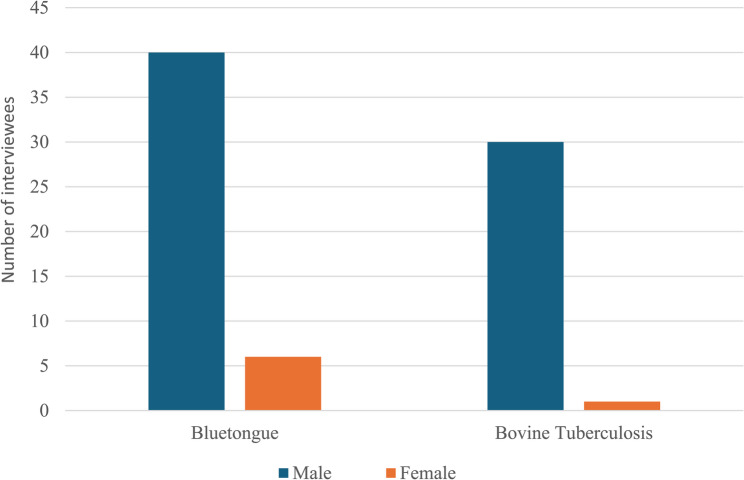
Number of male and female interviewees by topic of semi-structured interview

**Fig. 4:**
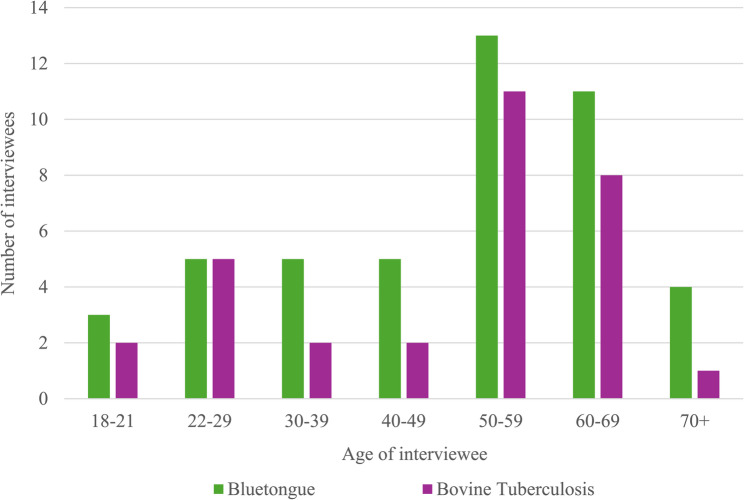
Age distribution of interviewees by topic of semi-structured interview

**Fig. 5:**
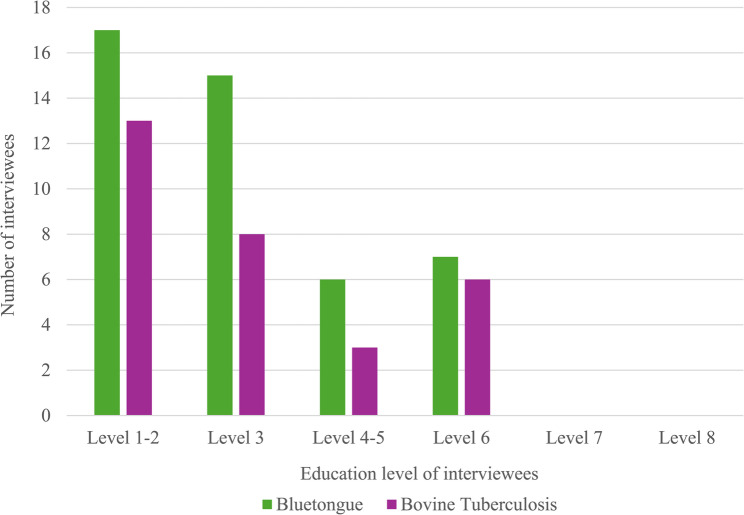
Education level of interviewees by topic of semi-structured interview. Definition of levels [19]. Level 1-2: GCSE, level 1 essential skills, level 1 functional skills, certificate level 1 or 2, award level 1 or 2, NVQ level 1 or 2, level 2 national certificate, level 2 national diploma, level 2 diploma, intermediate apprenticeship, ESOL level 1 or 2, or equivalent. Level 3: A level, AS level, NVQ level 3, advanced apprenticeship, international Baccalaureate diploma, level 3 award, level 3 certificate, level 3 diploma, level 3 ESOL, level 3 national certificate, level 3 national diploma, or equivalent. Level 4-5: Certificate or diploma of higher education, higher apprenticeship, higher national certificate or diploma, level 4 or 5 award, level 4 or 5 certificate, level 4 or 5 diploma, level 4 or 5 NVQ, foundation degree or equivalent. Level 6: Degree apprenticeship, degree, graduate diploma, level 6 award, level 6 certificate, level 6 diploma, level 6 NVQ, or equivalent. Level 7: Master's degree, level 7 award, level 7 certificate, level 7 diploma, level 7 NVQ, postgraduate certificate, PGCE, postgraduate diploma, or equivalent. Level 8: Doctorate, level 8 award, level 8 certificate, level 8 diploma, or equivalent.

In line with the interviews, respondents to the question slips were keenly aware of the limitations of the bTB skin test; in particular the potential for a herd to still be infected despite two clear whole herd tests. However, despite two identical questions regarding the reliability of bTB skin test being posed, the response of participants varied depending on the situation. Respondents had a lower level of confidence in a herd being truly free of bTB after two clear herd skin tests when it was a herd that they were purchasing from (Fig. 1) compared to when it was their own herd (Fig. 2). It is known from other fields of study that, despite accurate knowledge about the risks of a situation, people often underestimate their own risk.^2^  

### Bluetongue

Most farmers expressed concern about the risk of a BT incursion into NI:

“People in England tell me that it keeps on moving up the country, and we are scared that it could come across here.”

Sometimes these fears were abstract with statements simply expressing a desire to “keep it out” because “it’s a nightmare” whilst others were more specific referring to the potential impact that an incursion would have on trade:

“We want to keep it out of Ireland. We’re exporting to every country in Europe at the minute, and it’s leaving beef and sheep good money.”

or generations of breeding and the potential loss of genetics:

“Well, it’s scary because we are a wee island on our own and we have well established flocks and it would just decimate them.”

However, the clinical impact of a BT incursion was rarely directly mentioned. The potential impact of the disease was perceived in terms of government-imposed control measures, such as loss of export or culling.

All farmers spoke about the impact of government control measures to reduce the risk of a BT incursion. The inability to move animals from GB posed the biggest impact due to the financial implications of animals “stranded in GB” and the inability to introduce new genetics to pedigree flocks/herds. However, most farmers were pragmatic or supportive of the movement restrictions.

“Like, it’s hard. Obviously, we want to take animals over and sell them so the fact that you can’t really, without a lot of quarantine and stuff, is an issue but I understand we don’t want Bluetongue here. We don’t want to bring it back, so it is what it is. We have to deal with it.”

and

“We had quite a few sheep imported from 2021 to 2023 and that’s very vital for this as a breed but, obviously, I appreciate, I would be supporting, it is frustrating we can’t get anything in because we need stuff across, but obviously it’s necessary.”

A small number of farmers expressed cynicism about the control measures and spoke at length about the negative impact of the cattle movement restrictions citing specific examples. The primary predictor of this attitude was being a cattle farmer.

Despite fears of an incursion, most farmers reported that they had made no changes to their practices to reduce their risk. Views on vaccination were also mixed with many saying that they would consult their vet:

“I would talk to my vet and, if he advised it, I would yes.”

they would look at the research to determine whether it was worthwhile:

“I wouldn’t vaccinate until I’ve seen the science.”

or wait to see what their neighbours did before deciding to vaccinate:

“It’s no use unless everyone vaccinates because… they’d probably end up culling the whole area, if my neighbour gets it, they could cull me. So, hard to know.”

However, respondents to the question slips had a more positive attitude to vaccination with 12 of the 13 responses saying they would vaccinate if it was necessary.

Despite many farmers saying they had *“heard nothing”* or they *“know nothing”* about BT, many demonstrated awareness of the government control measures and the primary mechanism for transmission, the midge, which are difficult to control. However, it may also reflect that epizootic diseases, like bTB, are seen as a “government problem” rather than an industry problem:

“Really up to the Department and how strict they keep it. If stocks not coming in, and they keep it out…. I don’t think we should get it.”

## Discussion

The current study has highlighted several interesting and useful insights into farmer attitudes towards epizootic and enzootic diseases. This includes the challenges of ensuring that accurate, trustworthy and timely information reaches the relevant stakeholders in an era where, despite information being available continuously through multiple platforms, misinformation and disinformation is abundant and increasingly widespread. Given the trend of previous decades - an increasing number of platforms available for sharing information and wider and faster phone networks and WiFi coverage – the challenge posed by misinformation and disinformation is likely to increase in the future. Therefore, this is an area that requires attention by policy makers and government communication teams when tackling complex diseases with potentially competing conspiracy theories. 

It is encouraging to know that farmers understand the limitations of the bTB skin test; in particular the implications of its moderate sensitivity. However, their confidence that a herd is truly bTB free after regaining officially bTB free (OTF) status appears to differ depending on the context. This is consistent with research into knowledge and attitudes to risk in other areas [18]. This phenomenon could impact behaviour when buying and selling animals which would, in turn, impact disease eradication efforts. Further research into this area would be highly beneficial to determine the extent of this variation in different situations and its impact on behaviour/practice because previous research has suggested that cattle movements in NI are responsible for 6.4% of breakdowns [8] and cattle were the primary driver for transmission within the TVR area [2, 9]. 

Interestingly, despite webinars, stakeholder meetings and articles in the farming press, the clinical impact of a BT incursion was rarely directly mentioned. The potential impact of the disease was perceived in terms of government-imposed control measures, such as loss of export or culling. This was surprising given the initial clinical findings reported from the Netherlands [13, 20, 21] and serves as a reminder of the importance of keeping control measures proportionate to the impact of the disease, as well as ensuring that communication includes “the why” of disease control as well as “the what”. 

When discussing the impact of BT and bTB, the responses of interviewees differed. Usually, BT elicited an acute, often abstract, fear from farmers. In comparison, when considering bTB farmers tended to focus on the financial burden and mental health impact of long-term control measures. At the time of writing, NI has never had a BT incursion. Therefore, for many farmers, BT represents ‘the great unknown’, both in terms of when it will arrive, but also the impact it will have on their flock/herd and their business. As a result, their statements tended to be more abstract. In contrast, many farmer’s have been personally affected by bTB, or have relatives, neighbours or friends who have been, so their perception is based on real life experiences resulting in concrete statements regarding impact. Farmers were also more likely to have taken practical steps to reduce the risk of a bTB breakdown, such as raising water troughs or changing their buying-in policy, compared to a BT incursion which they often felt powerless to prevent. This lack of proactive, practical action may reflect the challenges of controlling BT. Many peer-reviewed papers report conflicting results for the use of insecticides as a control measure for BT [4, 10] and the current BTV-3 vaccination reduces, but does not prevent, viraemia. For both BT and bTB, the onus appeared to be on government bodies to control the disease but there were higher levels of trust and co-operation voiced by interviewees when discussing BT compared to bTB. However, there were a small number of farmers that expressed cynicism about the BT control measures and cited specific examples. These were primarily cattle farmers, but it is unclear why. It may simply reflect the higher value of cattle, compared to sheep, and thus the greater financial impact of BT movement restrictions. Alternatively, given that specific examples were cited, it may reflect that there was a cattle sale immediately prior to the introduction of restrictions meaning that cattle were more likely to be “stranded in GB” than sheep. In addition, in contrast to bTB, information regarding BT did not seem to be subject to conspiracy theories. Cattle farmers are more likely to have experienced bTB than sheep farmers, which could have resulted in their cynicism regarding bTB control measures influencing their perception of BT control measures.

The number of responses to individual question slips on the DAERA stand were low, with a maximum of 30 responses for a question. This means that the confidence intervals around point estimates are wide. Members of the public visiting the DAERA stand at the Balmoral Show could also complete multiple question slips. This means that the number of participants could be less than the number of responses to the question slips. In addition, visitors could select a question of interest to them. Whilst this was excellent for facilitating conversations with staff manning the DAERA stand, it does introduce bias as people with strong opinions on a subject may be more likely to select a question relating to that topic or particularly controversial topics may be avoided altogether. As a result, care in interpretation is required especially on controversial issues, such as wildlife control, compensation or BTV vaccination. It is important to ensure that the views elicited and reported truly reflect the population surveyed and not a minority that participate and choose a specific topic of interest. Therefore, further studies are required to provide an accurate profile on issues, such as wildlife interventions and compensation. In addition, due to the limited resource allocated to interviewing farmers, saturation was not reached [16]. This means that some topics, such as the perception of the role of government and governments vets in the control of infectious diseases or different attitudes towards BT movement controls among cattle farmers compared to sheep farmers, were not fully explored and definitive conclusions cannot be drawn.

## Conclusions

Previous research has highlighted the impact that prolonged eradication programmes without apparent progress, such as bTB, can have on morale and thus co-operation of farmers [6, 3, 12]. However, the current study also identified that in this environment conspiracy theories and superstitions can prosper. The advent of the internet and social media means that information and news are continuously available. However, incorrect and misleading information is also abundant. Ensuring that accurate information is disseminated in a timely fashion, and making sure it gains a stronger foot hold than the misinformation and disinformation, will become increasingly challenging, but necessary, for an eradication programme to be successful.

The limitations of the bTB skin test are well-known by farmers. Whilst the sample size was small, it appears that farmers’ confidence in the skin test varies depending on the situation. Determining the extent of this variation, in different contexts, and understanding how it impacts their behaviour, particularly when buying and selling cattle, would be beneficial to disease control policy. This warrants further research using qualitative research techniques and the use of games [14].

Whilst the interviews did not reach saturation on some topics, conversations regarding the impact of BT and bTB differed. BT elicited an acute, often abstract, fear from farmers. In comparison, the impact of bTB included the financial burden and mental health impact of a bTB breakdown.

The farming communities’ perception of the control policies for BT is, primarily, positive and well understood. However, the impact tended to be viewed in terms of the impact of control policies rather than clinical disease. It is important to ensure that the impact of control policies do not exceed the clinical impact of a BT incursion.

## Figures and Tables

**Fig. 6:**
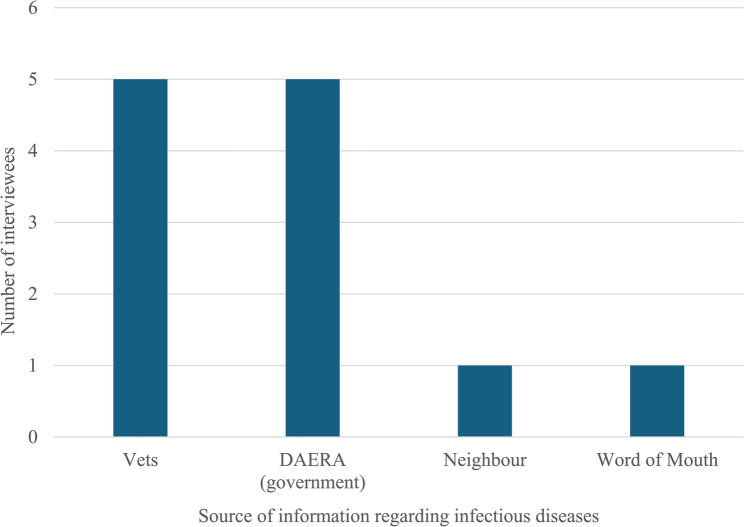
Responses to question; “If you have a bTB breakdown, where do you get your information from?” (*n* = 12)

## Data Availability

The datasets used and/or analysed during the current study are available from the corresponding author on reasonable request.

## Appendix

**Table 1:** Questions contained on the printed slips at the DAERA stand (*N*= number of responses*)

Bluetongue Questions	Bovine Tuberculosis Questions
When there is a disease outbreak, such as Bluetongue, where do you get information and why? (*N* = 0)	If you have a bTB breakdown, where do you get information from and why? (*N* = 12)
Do you think Northern Ireland will have an outbreak of Bluetongue this year? (*N* = 17)	If on-farm biosecurity advisory visits were available, would you be interested in participating? (*N* = 8)
If an emergency Bluetongue vaccine was made available, would you vaccinate your animals? (*N* = 13)	If you were told that an inconclusive reactor at a bTB test was 10 times more likely to have bTB than an animal that tests negative, what would you do with the inconclusive? (*N* = 0)
If your herd/flock was infected with Bluetongue, what impact on production do you think it would have? (*N* = 12)	If you were given a choice between the following three bTB control measures, (1) vaccinating badgers, (2) culling badgers or (3) testing badgers and then, based on the test result, choosing to vaccinate or cull, which would you prefer and why? (*N* = 30)
If you had Bluetongue in your herd/flock, what clinical signs would you expect to see? (*N* = 0)	If there was a badger intervention as part of the bTB control program, would you be willing to pay for it? How much would you be willing to pay per year? (*N* = 9)
	Do you think it is possible to eradicate bTB without a cattle vaccination? (*N* = 0)
	If you have a bTB breakdown, then have two clear, whole herd tests and regain bTB freedom, what do you think the probability is that your herd is bTB free? (*N* = 10)
	If you are buying an animal from an officially bTB free herd that had a breakdown in the previous 12 months, what do you think the probability is that the herd is bTB free? (*N* = 8)
	If you were DAERA’s CVO, what would you do to eradicate bTB? (*N* = 12)
	Do you feel able to make decision that will reduce the risk of a bTB breakdown in your herd? (*N* = 4)
	If you received higher compensation per reactor for implementing specific biosecurity measures, would you change biosecurity practices on your farm? How much additional compensation per animal would be required to: - Fence off badger setts - Prevent badgers accessing water troughs - Prevent badgers accessing feed stores - Isolate reactors - Double fence boundaries - Purchase replacements direct from farm - Become a closed herd - Only purchase from herds bTB free for more than 3 years. (*N* = 4)

* The number of responses to each question varies because participation was voluntary

**Table 2:** Number and length of interviews by topic and interviewer

Topic	Bluetongue	Bovine Tuberculosis	Total
Number of interviews Interviewer 1 Interviewer 2	46 24 22	31 11 20	77 35 42
Average length (mins) Interviewer 1 Interviewer 2	2.06 1.15 3.04	3.27 3.08 3.27	2.39 1.51 3.20

## Footnotes

1 In the context of a bTB breakdown after purchasing animals that had recently tested negative in the previous herd but tested positive once in their herd.

2 For example, if asked the probability of getting divorced, most individuals will correctly respond 50%. However, if asked the probability that they will get divorced themselves, most will quote a value less than 50% showing an unfounded optimism [18]. For health-related risks, such as cancer or heart disease, this unfounded optimism may negatively impact risk taking behaviour. For example, they may continue to smoke, or maintain an unhealthy diet etc.

## References

[1] Abernethy DA, Upton P, Higgins IM, McGrath G, Goodchild AV, Rolfe SJ, Broughan JM, Downs SH, Clifton-Hadley R, Menzies FD, de la Rua-Domenech R, Blissitt MJ, Duignan A, More SJ. Bovine tuberculosis trends in the UK and the Republic of Ireland, 1995–2010. Vet Rec. 2013. 10.1136/vr.100969. 23292950 10.1136/vr.100969

[2] Akhmetova A, Guerrero J, McAdam P, Salvador LCM, Crispell J, Lavery J, Presho E, Kao RR, Biek R, Menzies F, Trimble N, Harwood R, Pepler PT, Oravcova K, Graham J, Skuce R, du Plessis L, Thompson S, Wright L, Byrne AW, Allen A, Adrian R. Genomic epidemiology of Mycobacterium Bovis infection in sympatric Badger and cattle populations in Northern Ireland. Microb Genom. 2023. 10.1099/mgen.0.001023. 37227264 10.1099/mgen.0.001023 PMC10272874

[3] Biesheuvel MM, Santman-Berends IMGA, Barkema HW, Ritter C, Berezowski J, Guelbenzu M, Kaler J. Understanding farmers’ behavior and their Decision-Making process in the context of cattle diseases: A review of theories and approaches. Front Vet Sci. 2021. 10.3389/fvets.2021.687699. 34926632 10.3389/fvets.2021.687699 PMC8674677

[4] Carpenter S, Mellor PS, Toor SJ. Control techniques for Culicoides biting midges and their application in the UK and Northwestern palaearctic. Med Vet Entomol. 2008. 10.1111/j.1365-2915.2008.00743.x. 18816267 10.1111/j.1365-2915.2008.00743.x

[5] Chapman AL, Hadfield M, Chapman CJ. Qualitative research in healthcare: an introduction to grounded theory using thematic analysis. J R Coll Physicians Edinb. 2015. 10.4997/JRCPE.2015.305. 26517098 10.4997/JRCPE.2015.305

[6] Ciaravino G, Ibarra P, Casal E, Lopez S, Espluga J, Casal J, Napp S, Allepuz A. Farmer and veterinarian attitudes towards the bovine tuberculosis eradication programme in spain: what is going on in the field? Front Vet Sci. 2017. 10.3389/fvets.2017.00202. 29230403 10.3389/fvets.2017.00202 PMC5712013

[7] DAERA. CVO review of bovine tuberculosis in Northern Ireland November. 2024. Chief Veterinary Officer Review of Bovine Tuberculosis in Northern Ireland November 2024 | Department of Agriculture, Environment and Rural Affairs. Accessed 26th Aug 2025.

[8] Doyle LP, Courcier EA, Gordon AW, O’Hagan MJH, Stegeman JA, Menzies FD. Bovine tuberculosis in Northern ireland: quantification of the population disease-level effect from cattle leaving herds detected as a source of infection. Epidemiol Infect. 2017. 10.1017/S0950268817002424. 29103398 10.1017/S0950268817002424 PMC9148749

[9] Doyle LP, Gordon AW, Molloy C, O’Hagan MJH, Georgaki A, Courcier EA, Harwood RG, Menzies FD. Assessing the impact of a test and vaccinate or remove Badger intervention project on bovine tuberculosis levels in cattle herds. Epidemiol Infect. 2023. 10.1017/S0950268823001061. 37400974 10.1017/S0950268823001061 PMC10369427

[10] EFSA Panel on Animal Health and Welfare. Bluetongue: control, surveillance and safe movement of animals. EFSA J. 2017. 10.2903/j.efsa.2017.4698. 10.2903/j.efsa.2017.4698 PMC7009973 32625424

[11] Georgaki A, Murchie A, McKeown I, Mercer D, Millington S, Thurston W, Burns K, Cunningham., Harkin V, Menzies FM. Bluetongue disease control in Northern Ireland during 2017 and 2018. Front Vet Sci. 2019. 10.3389/fvets.2019.00456. 31921914 10.3389/fvets.2019.00456 PMC6928110

[12] Hamilton L, Evans N, Allcock J. I don’t go to meetings: Understanding farmer perspectives on bovine TB and biosecurity training. Vet Rec. 2019;184(14):410. 10.1136/vr.104995. 30617111 10.1136/vr.104995

[13] Santman-Berends IMGA, van den Brink KMJA, Dijkstra E, van Schaik G, Spierenburg MAH, van den Brom R. The impact of the bluetongue serotype 3 outbreak on sheep and goat mortality in the Netherlands in 2023. Prev Vet Med. 2024. 10.1016/j.prevetmed.2024.106289. 39126984 10.1016/j.prevetmed.2024.106289

[14] Hiteva R, Barron A, Pottinger L. Methods for change: playing games as method. Aspect network. 2021. Ralitsa-Hiteva-A4-Guide-05-Oct.pdf. Accessed 19th Sept 2025.

[15] Menzies FD, McCullough SJ, McKeown IM, Forster JL, Jess S, Batten C, Murchie AK, Gloster J, Fallows JG, Pelgrim W, Mellor PS. Oura C.A.L. Evidence for both transplacental and contact transmission of bluetongue virus in cattle. Vet Rec. 2008. 10.1136/vr.163.7.203. 18708653 10.1136/vr.163.7.203

[16] Saunders B, Sim J, Kingstone T, Baker S, Waterfield J, Bartlam B, Burroughs H, Jinks C. Saturation in qualitative research: exploring its conceptualization and operationalization. Qual Quant. 2018. 10.1007/s11135-017-0574-8. 29937585 10.1007/s11135-017-0574-8 PMC5993836

[17] Smith W, McCann J, Philimore A, Jones L. Sept, Finding the box-top: addressing the human cost of bTB. Farming Community Network. 2025. bTB-report-2025-final-Addressing-the-human-cost-of-bTB.pdf. Accessed 17th 2025.

[18] Thaler RH, Sunstein CR. BChapter 1:Biases and Blunders. In: Nudge. Penguin Book Ltd. 2021. pp. 32–34.

[19] UK Government. What qualification levels mean. 2025. What qualification levels mean: England, Wales and Northern Ireland - GOV.UK. Accessed 18th Sept 2025.

[20] van den Brink KMJA, Santman-Berends IMGA, Harkema L, Scherpenzeel CGM, Dijkstra E, Bisschop PIH, Peterson K, van de Burgwal NS, Waldeck HWF, Dijkstra T, Holwerda M, Spierenburg MAH, van den Brom R. Bluetongue virus serotype 3 in ruminants in the netherlands: clinical signs, Seroprevalence and pathological findings. Vet Rec. 2024. 10.1002/vetr.4533. 39148262 10.1002/vetr.4533

[21] van den Brink KMJA, Brouwer-Middelesch H, van Schaik G, Lam TJGM, Stegeman JA, van den Brom R, Spierenburg MAH, Santman-Berends IMGA. The impact of bluetongue serotype 3 on cattle mortality, abortions and premature births in the Netherlands in the first year of the epidemic. Prev Vet Med. 2025. 10.1016/j.prevetmed.2025.106493. 40054335 10.1016/j.prevetmed.2025.106493

[22] Williams M, Moser T. The Art of coding and thematic exploration in qualitative research. Int Manag Rev. 2019;15:45–55.

